# Lived experiences and coping strategies among cancer patients in the Volta Region of Ghana: A health facility-based qualitative study

**DOI:** 10.1371/journal.pmen.0000310

**Published:** 2025-04-30

**Authors:** Emmanuel Abu Bonsra, Leslie Kwesi Morrison, Haddiyat Offeibea Anderson, Mark Kwame Ananga, Hubert Amu

**Affiliations:** Department of Population and Behavioural Sciences, Fred N. Binka School of Public Health, University of Health and Allied Sciences, Hohoe, Ghana; University of Turin, ITALY

## Abstract

Cancer poses a major global health challenge, with significant emotional and psychological impacts on patients. In Ghana, limited mental health support for cancer patients remains a concern. This study explores the lived experiences, coping strategies, and mental health service availability among cancer patients. This qualitative exploratory study recruited 20 cancer patients and 9 health professionals from Ho Teaching Hospital. Data were collected using in-depth interviews and analysed thematically with Atlas.ti 7.5.7. Regarding the lived experiences of cancer patients, the study unveiled profound emotional impact of receiving a cancer diagnosis, leading to a sense of devastation, fear, and even suicidal ideation among patients. Ongoing emotional challenges, including persistent depression, treatment-induced stress, and constant anxiety were prevalent throughout the cancer journey. Coping strategies adopted by patients included seeking support from loved ones, immersing themselves in work and avoidance-focused coping mechanisms, such as isolation and denial. Specialized mental health support was largely unavailable, but nurses played a critical role in providing emotional support. Cancer patients experience profound emotional distress, highlighting the need for integrated mental health services. Addressing stigma, service gaps, and workforce shortages could enhance patient well-being and support Sustainable Development Goals (SDGs), particularly SDG 3 (‘Good Health and Well-being’) and SDG 10 (‘Reduced Inequalities’). The Ghana Health Service and the Ministry of Health should increase mental health funding, expand professional training, and raise public awareness to bridge these gaps and improve holistic cancer care.

## Summary

This study investigates the emotional experiences and coping strategies of cancer patients in the Volta Region of Ghana, focusing on those receiving treatment at Ho Teaching Hospital. Through in-depth interviews with 20 cancer patients and 9 healthcare professionals, we explored the challenges patients face during their cancer journey, along with the availability of mental health services. Our findings reveal that cancer patients experience significant emotional distress, including shock, fear, depression, and suicidal ideation. Many rely on informal coping strategies such as seeking support from family and friends, prayer, and engaging in music and movies. However, the lack of structured mental health services both at the hospital and within the community exacerbates their struggles. The study underscores the importance of integrating mental health care into cancer treatment and ensuring that patients have access to emotional and psychological support. It also highlights the need for healthcare systems in resource-limited settings to address the mental health needs of cancer patients to improve their well-being and treatment outcomes. Ghana can make significant progress toward achieving Sustainable Development Goals (SDG 3: Good Health and Well-being, and SDG 10: Reduced Inequalities). However, the study emphasizes the need for concrete policy and practice changes to make mental health services more accessible and effective for cancer patients in the region.

## Introduction

### Background

Cancer is a multifaceted disease that, if not detected and managed promptly, can often lead to fatal outcomes [1]. In 2015, cancer was responsible for over 8.7 million deaths globally, making it the second leading cause of death after cardiovascular diseases [2]. While cancer significantly impacts physical health, it also contributes to mental health challenges, including psychological distress, anxiety, and depression [2]. These conditions can negatively affect patients’ quality of life and overall treatment outcomes. One of the severe mental health concerns among cancer patients is suicidal behaviour, which includes suicidal ideation (SI), suicide attempts (SAs), and completed suicides [3]. Suicide represents a significant public health challenge worldwide, being the leading cause of violent death across populations [3]. As cancer remains a leading cause of death with increasing incidence rates globally [3], it is associated with severe psychological distress and psychiatric symptoms, particularly depression [2,4], which can further elevate the risk of suicide [5]. Indeed, cancer is recognized as a contributing factor to increased suicide risk [6]. Understanding how physical and psychological stressors relate to suicidal behaviour in cancer patients is a crucial goal of oncological research, with potential implications for preventive strategies [6]. However, it is essential to distinguish general mental health concerns from suicidality, as not all cancer patients experiencing distress develop suicidal thoughts or behaviours [6].

Receiving a cancer diagnosis is often a traumatic event that can trigger immediate adverse health effects beyond the disease or its treatment [4]. Cancer patients, particularly those with advanced stages of the illness, are notably susceptible to suicidal ideation (SI) and suicide attempts (SAs) [3,7]. Recent cancer diagnoses have been linked to increased risks of both suicide and death from cardiovascular causes compared to individuals without cancer [2]. Effective management of this crisis period hinges on psychological factors, including the patient’s prior adjustment capacity, potential for rehabilitation, personality traits, coping mechanisms, and the influence of cultural and religious beliefs, in addition to social support systems [8]. Several studies in high-income countries have explored the intersection between cancer and mental health, highlighting the psychological burden cancer patients endure and the coping strategies they adopt [9]. Research has consistently shown that access to psychosocial support services can significantly reduce emotional distress and improve patients’ quality of life [8]. However, there is a limited body of research focusing on the mental health challenges faced by cancer patients in sub-Saharan Africa, particularly in Ghana. While studies in Ghana have examined general cancer burden and treatment outcomes [2], there is a gap in the literature concerning the psychological distress, suicidal behaviour, and coping strategies of cancer patients in this context.

In Ghana, the suicide rate was estimated to be approximately 6.6 per 100,000 population in 2019, according to World Bank development indicators [9]. Annually, around 1,993 suicides are reported in the country [10]. Specifically, in northern Ghana, a study indicated a prevalence rate of 3.94% for suicidal deaths, with common methods including hanging and poisoning [11]. Despite the increasing recognition of these issues, there is a significant gap in the literature concerning the experiences of suicidal behaviour and coping strategies among cancer patients at Ho Teaching Hospital. The absence of studies on this subject creates a critical gap in understanding the extent of psychological distress and the availability of mental health support for this vulnerable group. Despite the growing burden of cancer in Ghana, mental health services remain underdeveloped, and psychosocial support is often inadequate. Existing studies in Ghana primarily focus on cancer treatment and mortality rates but do not sufficiently explore the emotional and psychological experiences of cancer patients. Furthermore, suicidal ideation and coping mechanisms among this population remain underexplored. Given that mental health care is not fully integrated into oncology services in many Ghanaian hospitals, understanding the lived experiences of cancer patients is crucial for developing patient-centered interventions. Findings from this study will provide essential insights that can inform healthcare policies, enhance mental health integration into oncology care, and contribute to the global discourse on cancer-related mental health challenges in low-resource settings. This study aims to explore the lived experiences of cancer patients at Ho Teaching Hospital, assess the coping strategies they employ in managing mental health challenges related to cancer, and evaluate the current mental health services and resources available to them.

### Theoretical issues

The current study is grounded in the Interactive Stress Theory, developed by American psychologist Thomas Joiner in the 1990s [12]. This theory focuses on the interplay between stress, coping mechanisms, and mental health outcomes, particularly in the context of suicidal behaviour and depression. It provides a framework for understanding how individual, environmental, and situational factors contribute to suicide risk.

According to the theory, suicide results from a combination of three critical factors: (1) a sense of being a burden to others (individual level), (2) feelings of loneliness and social isolation (environmental level), and (3) the acquired capability for self-harm (situational stressors). These factors interact with each other and with additional life stressors, further heightening the risk of suicidal behaviour.

Environmental factors, such as lack of social support or financial strain, can intensify feelings of loneliness and isolation. Situational factors, including the stage and progression of cancer, can impact an individual’s physical and emotional well-being. Additionally, the acquired capability for self-harm—developed through repeated exposure to physical or emotional pain—can increase an individual’s risk of suicide. This study explores whether cancer patients have developed a higher pain tolerance and how this influences their risk of suicidal behaviours. Self-report measures, clinical assessments, and other relevant data will be used to examine these factors.

**Fig 1:**
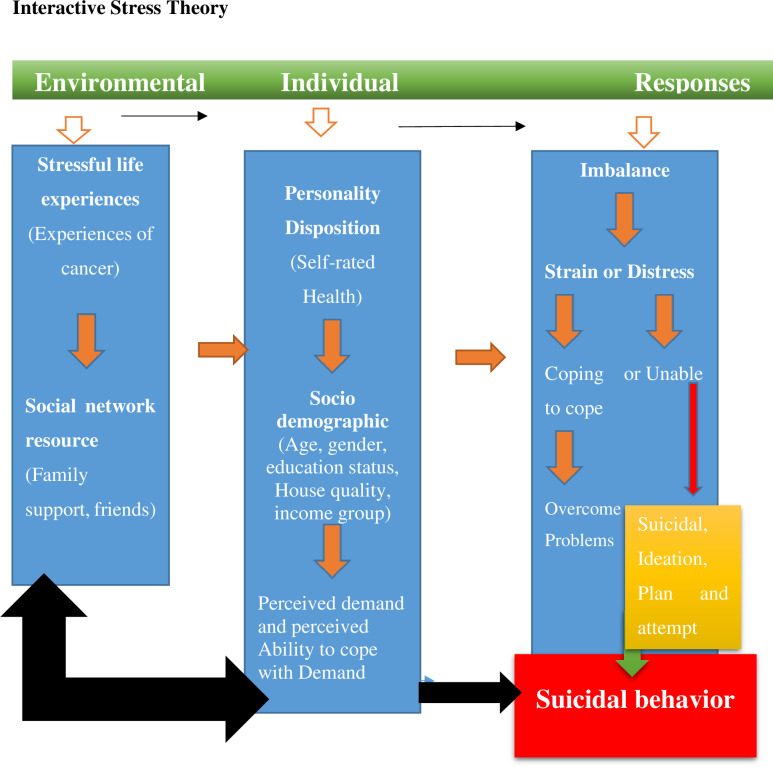
Interactive stress theory framework of the study.

The interplay of these elements underscores the significance of understanding how stress, social factors, and illness progression contribute to suicidal risk among cancer patients. By applying the Interactive Stress Theory, this study aims to provide insights into the complex interactions that may increase vulnerability to suicide in this population (Fig 1).

## Methods and materials

### Ethics statement

Ethical clearance was obtained from the University of Health and Allied Sciences Review Ethics Committee (UHAS-REC A.10 [103]22–23) and the Institutional Review Boards of the Ho Teaching Hospital research department. Permission was also sought from department and centre heads at the hospitals before data collection commenced. Informed consent was obtained from participants after providing detailed information about the study’s objectives, procedures, potential risks, and benefits in a language they understood. The consent process ensured that participants were aware of their right to withdraw from the study at any time without any repercussions on their care. Participants were provided with consent forms to sign or thumbprint after the information session, and a witness was present for participants with literacy challenges. To safeguard participant confidentiality, pseudonyms were used instead of real names and other identifying characteristics. Data security was prioritized: audio recordings were encrypted and stored using a password-protected program called ‘My Lockbox,’ while typed notes were securely stored in the same system. Physical copies of data were kept in locked cabinets accessible only to the research team.

Given the sensitive nature of the study, extra measures were implemented to ensure participants’ emotional well-being. During the consent process, participants were informed about the potential for emotional distress during interviews and reassured that a trained counsellor would be available on-site to provide immediate support if needed. Participants were also given the option to pause or stop the interview at any point without penalty. Interviews were structured to conclude with less emotionally charged questions to help participants transition out of any distress. Additionally, participants received contact information for follow-up mental health support and were assured of a post-interview check-in to monitor their well-being. These measures underscored the research team’s commitment to minimizing harm and supporting participants throughout the study.

### Study setting

The study was conducted at Ho Teaching Hospital, one of the five public teaching hospitals in Ghana. Initially constructed by Kaevener Construction International of the United Kingdom, the hospital was completed and handed over to the Government of Ghana in November 1998. It began operations on a small scale in April 1999 and was officially commissioned as the Volta Regional Hospital in December 2000 by former President John Jerry Rawlings and his wife. Strategically located, the hospital provides specialized healthcare services to the Volta Region and neighbouring countries such as Togo, Benin, and Nigeria. It envisions itself as a Medical Tourist Centre, focusing on tertiary healthcare, medical education, and research.

### Study design

This study adopted a qualitative exploratory design to examine the lived experiences and coping strategies of cancer patients at Ho Teaching Hospital in the Volta Region of Ghana. This approach allowed for an in-depth exploration of the psychological, emotional, and social challenges faced by participants. In-depth interviews were used to collect detailed narratives, enabling participants to share their perspectives freely. The qualitative design ensured a comprehensive understanding of the themes emerging from their experiences, providing valuable insights into coping mechanisms and mental health challenges in the context of cancer.

### Study population

The primary study population comprised adult cancer patients receiving care at the Ho Teaching Hospital and healthcare professionals from the hospital’s oncology department. Cancer patients were selected based on their diagnosis and treatment at the hospital, while healthcare professionals included oncologists, nurses, and social workers involved in the care of these patients.

### Inclusion criteria

All adults (18 years or older) with a confirmed cancer diagnosis, the ability to understand and provide informed consent, and who resided in the Volta Region and received care at the Ho Teaching Hospital, were included in this study. Additionally, healthcare workers who directly interact with cancer patients were involved in the study

### Exclusion criteria

Cancer patients with a severe mental illness or disability affecting their ability to participate in the study and below 18years were excluded. Additionally, healthcare professionals who do not directly deal with cancer patients were also excluded.

### Sampling procedure

A purposive sampling technique was used to recruit adult cancer patients and healthcare professionals. Data saturation was reached with twenty cancer patients and nine healthcare professionals. Patients were recruited in collaboration with the hospital’s oncology department, ensuring diversity in cancer types and stages. Healthcare professionals, including oncologists, nurses, and social workers, were selected based on their direct interaction with cancer patients and their varying levels of experience and expertise.

### Sampling size determinants

Initially, forty-four participants were approached for the study; however, nine patients and six healthcare professionals declined to participate due to illness, communication difficulties, or time constraints. One interview with a healthcare professional was excluded as it was interrupted by an emergency. The final sample size of 20 cancer patients and 9 healthcare professionals was determined based on data saturation, a widely accepted principle in qualitative research. Data saturation occurs when no new themes or insights emerge from additional interviews, making further data collection redundant. Previous qualitative studies have demonstrated that saturation is typically achieved within 12–20 interviews in homogeneous populations. The inclusion of healthcare professionals ensures diverse perspectives on patient experiences and available support systems, enhancing the study’s depth and validity.

### Research team

Data collection was carried out by three researchers: the first author (male), the second author (male), and the third author (female). All team members had prior experience with qualitative interviews. A two-day training session ensured consistency in the use of research instruments and interview techniques. The recruitment period for this study started on 10th June 2023 and ended on November 30, 2023.

### Data collection procedure

In-depth, face-to-face interviews were conducted with both cancer patients and healthcare professionals at Ho Teaching Hospital. Data collection involved in-depth interview guides and structured questionnaires with cancer patients and healthcare professionals to gather comprehensive insights into the emotional experiences, coping strategies of cancer patients, and the availability and accessibility of mental health services from the perspective of healthcare professionals.

### Cancer patients

Cancer patients were interviewed to explore their perceptions of the emotional challenges associated with their diagnosis, the coping mechanisms they employ, and the mental health support they have received. Interviews were conducted in a private setting to ensure confidentiality and comfort, with the option for patients to be interviewed in English or local languages (Ewe, Twi, and Hausa). Interviews with patients typically lasted about 35 minutes. To ensure accurate data capture, both audio recordings and handwritten notes were made with the participants’ consent. The research team was trained to handle sensitive topics, ensuring that any distress experienced during the interview was addressed appropriately.

### Healthcare professionals

Healthcare professionals, including oncologists, nurses, and social workers, were interviewed to gain insights into the availability and accessibility of mental health services for cancer patients. These professionals were selected based on their direct involvement in patient care. The interviews with healthcare professionals were conducted in English and lasted approximately 25 minutes. The focus of the interviews was on identifying gaps in mental health support, exploring current practices in addressing patients’ emotional and psychological needs, and understanding the challenges in providing mental health services to cancer patients.

### Recruitment period

The recruitment period for this study started on June 10, 2023, and ended on November 30, 2023. Audio recordings and handwritten notes were utilized to prevent data loss due to potential equipment failure. Participants were informed that the study was part of the researchers’ undergraduate academic requirements. Two separate interview guides were used, tailored to cancer patients and healthcare professionals.

### Challenges encountered

Challenges included patients’ health conditions affecting their ability to participate, time constraints due to medical appointments, and an instance where a health professional had to leave an interview to address an emergency. Flexible scheduling and sensitivity to participants’ needs were adopted to mitigate these challenges.

### Trustworthiness

To ensure the trustworthiness of this qualitative study, we adhered to the principles outlined by Korstjens and Moser, which emphasize four essential **psychometric indicators**: credibility, transferability, dependability, and confirmability [13]. Each of these indicators was rigorously addressed throughout the research process to ensure the validity, reliability, and authenticity of our findings.

### Credibility

Credibility refers to the accuracy and believability of the findings. To ensure credibility, we employed several triangulation techniques:

- **Data Triangulation**: We collected data from multiple sources, including cancer patients and healthcare professionals. By comparing insights from these different perspectives, we could validate key themes and issues related to Lived experience, suicidal behaviour and coping strategies. This also helped to identify patterns of consistency across different groups.
- **Investigator Triangulation**: Three independent researchers (the first, second, and third authors) were involved in the coding and theme development process. Each researcher developed their initial codes and themes independently and then came together to discuss and refine them. This process minimized individual bias and enhanced the depth of analysis by incorporating multiple viewpoints and interpretations.
- **Member Checking**: While we did not return transcripts to participants for verification due to logistical constraints, the research team ensured that the interview data were accurate by cross-checking the transcriptions with audio recordings and handwritten notes. This helped to ensure the data’s credibility before analysis.

### Transferability

Transferability refers to the extent to which the findings can be applied to other settings or populations. To ensure transferability, we provided a comprehensive description of the study context and participant demographics, which included detailed information about the setting, the hospital, the target population, and the sampling methods. We also included:

- **Contextual Information**: A thorough description of the Ho Teaching Hospital’s history, operations, and geographical setting was provided to help readers assess the transferability of the study findings to other hospital settings or healthcare environments.
- **Detailed Participant Demographics**: Information on the participants’ socio-demographic characteristics, including age, gender, education, and occupation, was outlined to give readers a better understanding of the sample’s diversity. This allows others to determine whether the findings are applicable to other cancer patient populations in different settings.
- **Thematic Tables and Interview Guides**: We provided thematic tables that summarize the key findings from the study, along with sample interview guides used for both patients and healthcare professionals. These tools give readers insight into the structure of the interviews and the types of questions asked, making it easier for them to understand how the study’s methods could be adapted to similar research contexts.

### Dependability

Dependability concerns the consistency and reliability of the study’s findings. To ensure dependability, we employed several strategies:

- **Documentation of the Research Process**: The entire research process, from study design to data collection and analysis, was meticulously documented. This documentation allows for transparency and accountability, making it easier for other researchers to follow the steps we took and understand the rationale behind our decisions.
- **Audit Trail**: An audit trail was maintained throughout the study, where all decisions regarding the research process (e.g., the inclusion of specific themes, data analysis procedures, or modifications to the interview guides) were carefully recorded. This allowed us to trace how the findings emerged from the data and ensured that the research process was both systematic and reproducible.
- **Codebook Development**: A codebook was created to guide the coding process. This codebook outlined the initial codes, their definitions, and the rules for applying them. The codebook helped ensure consistency in the coding process, allowing the research team to maintain dependability throughout data analysis.

### Confirmability

Confirmability relates to the extent to which the findings reflect the participants’ perspectives, rather than the researchers’ biases or preconceptions. To ensure confirmability, we took the following steps:

- **Reflexivity**: Reflexivity was a key component of our analysis process. The researchers-maintained awareness of their own biases, backgrounds, and preconceptions, and regularly reflected on how these might influence the research process and interpretation of findings. Personal biases were documented and actively monitored during data collection and analysis to ensure that the findings were grounded in the data rather than influenced by the researchers’ personal views.
- **Independent Coding**: To reduce researcher bias, coding was conducted independently by three researchers (ABE, MLK, and AOH), each of whom brought different perspectives to the data analysis process. Disagreements were resolved through discussion and consensus, and the final codes and themes were agreed upon by all team members.
- **Audit Trail and Transparency**: The audit trail mentioned under dependability was also important for confirmability, as it ensured that the conclusions drawn from the data were based on documented evidence. By clearly documenting the steps taken and the decisions made, we ensured that the results could be traced back to the raw data, minimizing the possibility of bias influencing the final findings.

By rigorously addressing these four dimensions—credibility, transferability, dependability, and confirmability—we sought to enhance the validity and reliability of our qualitative research. These steps ensured that the findings of this study offer a credible, transparent, and trustworthy exploration of the experiences of cancer patients and healthcare professionals in the context of suicidal behaviour and coping strategies at Ho Teaching Hospital

### Data analysis

Collected data were transcribed and proofread for accuracy. Non-English interviews were translated into English, and transcripts were cross-checked with handwritten notes and audio recordings to ensure fidelity. Due to logistical constraints, transcripts were not returned to participants for verification. Reflexive thematic analysis was conducted using both inductive and deductive approaches. Initially, an inductive approach was employed, where themes were derived directly from the data without predefined categories. This allowed for an open exploration of emerging patterns. Concurrently, a deductive approach was applied, informed by existing literature on suicidal behaviour and coping strategies among cancer patients, ensuring that relevant theoretical constructs were considered. Transcripts were read and re-read to ensure familiarity. Initial coding was performed by ABE, while MLK and AOH conducted independent confirmatory coding. A codebook was developed to organize codes systematically, and codes were then categorized into sub-themes and main themes based on shared patterns. Themes were refined through iterative discussions among the research team to ensure coherence and consistency. The final thematic structure was established collaboratively by EAB, MLK, and AOH. Thematic coding was conducted using ATLAS.ti version 7.5.7, allowing for systematic organization and analysis of qualitative data. To illustrate findings, direct quotations from respondents are presented, while frequency tables summarize participants’ socio-demographic characteristics.

## Results

We present the results of this study based on the themes from the analysis conducted. The background characteristics of the participants are also presented.

### Socio-demographic characteristics of participants

Table 1 presents the socio-demographic characteristics of the participants. Out of the total respondents (N = 20), 7 participants (35%) were aged 45 years, 5 (25%) were aged 52 years, 5 (25%) were aged 58 years, 2 (10%) were aged 59 years and 72 years 1(5%). In terms of sex, 4 participants (20%) were male, and 5 (25%) were female. Regarding ethnic background, 10 participants (50%) were Ewe, followed by 7 (35%) Akan and 3 (15%) Ga-Dangme. Most participants had completed Junior High School (JHS), with 13 (65%), while 7 (35%) had attended only basic school. The participants’ areas of residence were diverse, with 7 (35%) living in Awatidome, 5 (25%) in Ho, 5 (25%) in Donorkordzi, 2 (10%) in Tamale, and 1 (5%) in Cape Coast. The majority of participants were Christian, accounting for 13 (65%), while 7 (35%) were Muslim. In terms of marital status, most were separated (10, 50%), while 7 (35%) were married, and 3 (15%) were widowed. Employment status showed that the largest group were farmers 7(35%), followed by food vendors and retired/pensioners 5 (25%) respectively, 2(10%) engaged in tailoring and trading 1(5%). Additionally, 13 participants (65%) had health insurance, while 7 (35%) did not.

**Table 1:** Socio-demographic characteristics of patients.

Demographic features	Frequency (N = 20)	Percentage (%)
**Age**		
45	7	35.0
52	5	25.0
58	5	25.0
59	2	10.0
72	1	5.0
**Sex**		
Male	4	20.0
Female	5	25.0
**Ethnic Group**		
Akan	7	35.0
Ewe	10	50.0
Ga-Dangme	3	5.0
**Level of education**		
JHS	13	65.0
Basic school	7	35.0
**Area of residence**		
Awatidome	7	35.0
Ho	5	25.0
Donorkordzi	5	25.0
Tamale	2	10.0
Cape Coast	1	5.0
**Religion**		
Christian	13	65.0
Muslim	7	35.0
**Marital status**		
Married	7	35.0
Separated	10	50.0
Widowed	3	5.0
**Employment Status**		
Farmer	7	35.0
Food Vendor	5	25.0
Retired/ Pensioner	5	25.0
Tailor	2	10.0
Trader	1	5.0
**Ownership of Health insurance**		
Yes	13	65.0
No	7	35.0

### Socio-demographic features of the health professionals

Table 2 presents the socio-demographic characteristics of the health professionals involved in the study. Among the 9 participants, the majority were aged 34 years (66.6%), while 33.3% were aged 42 years. In in terms of sex 4 (44.4%) were male and 5 (55.5%) were female health professionals. Ethnically, most of the participants were Akan (66.6%), with 33.3% identifying as Ewe. Regarding education, the majority had completed a diploma (66.6%), while 33.3% had a degree. All participants resided in Ho, with 77.7% never married and 22.2% married. The staff ranks varied, with 44.4% being senior staff nurses and 55.5% general nurses. In terms of experience, 55.5% had been working as nurses for 1–5 years, and 44.4% had more than six years of experience. Similarly, 55.5% had worked at the health facility for 1–5 years, and 44.4% had worked there for more than six years.

**Table 2:** Socio-demographic characteristics of health professionals.

Variables	Frequency (N = 9)	Percent (%)
**Age**		
34	6	66.6
42	3	33.3
**Sex**		
Male	4	44.4
Female	5	55.5
**Ethnic Group**		
Akan	6	66.6
Ewe	3	33.3
**Level of education**		
Degree	3	33.3
Diploma	6	66.6
**Area of residence**		
Ho	9	100
**Region**		
Christian	3	33.3
Muslim	6	66.6
**Marital status**		
Never married	7	77.7
Married	2	22.2
**Rank**		
Senior Staff Nurse	4	44.4
General nurse	5	55.5
**Years of working as a nurse**		
One -five years	5	55.5
Above six years	4	44.4
**Years of working at a Health facility**		
One -five years	5	55.5
Above six years	4	44.4

### Thematic results

Table 3 presents the themes from our analysis: experiences, coping strategies, and the mental health services available to and for cancer patients. This table presents the thematic analysis from both patients and healthcare professionals. The thematic analysis, which focuses on the availability and accessibility of mental health services, solely focuses on the analysis from both patients and healthcare providers. These themes emerged through a reflexive thematic analysis, where transcripts were coded inductively to capture recurring patterns in participants’ narratives.

**Table 3:** Thematic table.

Main theme	Sub-theme	Codes
**Experiences**	**Initial Emotional Response**	◦Awful
		◦Heavy emotional blow
		◦hurt
		◦Shock
	**Ongoing Emotional Challenges**	◦Shock and Fear
		◦Suicidal ideation
		◦Persistent Depression
		◦Stress from Treatment
		◦Constant Anxiety
	**Evolving Emotional States**	◦Shock and Fear
		◦Feelings of Being a Burden
		◦Helplessness
		◦Lack of Support
		◦Moments of Hope
**Coping Strategies**	**Emotional Coping Strategies**	◦Seeking Support from Friends and Family
		◦Seeking Solace in Music and Movies
		◦immerse myself in my work
		◦Sleeping
	**Avoidance-Focused Coping**	◦Avoid thinking
		◦Initial Avoidance of Treatment
		◦Isolation
		◦Just to die
		◦Struggled with sleeping
	**Source of Coping Strategies**	◦Counselling at hospital
		◦Friends and family
		◦Mental Health professional
		◦Nurses
		◦Pastor
		◦Prayer and faith
	**Effectiveness of Coping Strategies**	◦condition remains same
		◦helped endure
		◦No significant improvement
		◦Psycho improvement
**Mental Health Services and Resources for Cancer Patients**	**Type of mental health support**	◦Counselling sessions
		◦No support received
		◦No service in the community
		◦No service received
		◦No specialized support
		◦No support group
	**Reasons for Lack of Mental Health Support**	◦Availability
		◦Stigma
	**Type of community services**	◦No services available

### Live experiences of cancer patients

According to the respondents, the initial emotional responses to receiving a cancer diagnosis were overwhelmingly characterized by a profound sense of devastation and distress, with most patients describing it as ‘awful’ and ‘a heavy emotional blow.’ In addition, the shock and fear associated with the diagnosis were commonly reported, often leading to the emergence of suicidal thoughts and plans, although many respondents noted their efforts to push these thoughts away. A 72-year-old male patient for instance noted

*“Yes oh…. Suicidal thoughts and the plan did come into my mind, but I’ve managed to push them away”.* Another patient, a 58-year-old male also stated: “*The diagnosis hit me hard at the age of 56. Suicidal thoughts did cross my mind, but I’ve found ways to push through though it is not easy at all my son”*.

Another patient said:

*‘’I was told about it the diagnosis was a heavy emotional blow. And I decided to kill myself. Suicidal thoughts were always in my mind, but I’ve managed to hold on’’* (Patient, female, 58 years).

A 45-year male, patient said*: “Oh my brother, mm…. the cancer diagnosis was awful, and I seriously considered suicide.”* Other Patient also said:

As the cancer journey develops, patients face ongoing emotional challenges, primarily characterized by persistent depression, stress induced by treatments, and constant anxiety. The burden of managing the disease and its associated treatments takes a toll on their emotional well-being. A patient said.

*‘’ The thing is I am always depressed, and sometimes too stress, and anxiety have been constant companions in my life now. Because sometimes going to the hospital for treatment is very difficult. I don’t have enough money for the treatment and even what to eat is a big problem now so am always depressed”*

(Patient, Female, 59 years)

According to the responses provided by the respondents, their emotional states have evolved significantly since the time of diagnosis, reflecting a dynamic and challenging emotional journey. Initially, they felt profound shock and fear upon receiving the diagnosis. As time passed, they grappled with the sense of being a burden to loved ones and experienced a persistent feeling of helplessness. The respondent also noted a recurring theme of perceived isolation and a lack of support from their social circle. Nevertheless, amidst these challenges, they described brief moments of hope, highlighting the resilience and emotional complexity inherent in the journey of living with cancer.

A female, 45 years stated:

*“I am a burden and helpless at times to my family. I’ve also felt that no one truly cares, and I always feel sad because people don’t come closer to me”.*

Other patients also had these to say

“*The news gave me a shock and scared me. It came to a point where I felt like a burden on my family, helpless, and mm …. There are times I believed no one cared, erh you see I am old now so if I die today, I will be happy”*

(Patient, Male, 72 years).

These findings highlight how the emotional burden of cancer extends beyond physical symptoms, influencing patients’ mental health and social relationships.

### Coping strategies adopted by cancer patients

Coping Strategies in the context of cancer patients were grouped into two main category sub-themes. The first, “Emotional Coping Strategies,” involves seeking support from loved ones, immersing in work, and finding solace in art and sleep. The second, “Avoidance-Focused Coping,” includes responses like denial, sleep difficulties, isolation, and avoidance of treatment. These strategies draw from various sources, including counselling, support networks, healthcare professionals, and faith. Their effectiveness varies, with some patients enduring their condition, while others experience no significant improvement or positive shifts in psychological well-being. Patients often employ various emotional coping strategies to address the challenges associated with their condition.

A patient stated clearly that:

“*Support from friends and family keeps me going. I find solace in gardening and spending time with nature by sitting in the chair under a tree in my house with my radio listening to music and news.”*

(Patient, Male, 58 years).

Other patients had these to say:

“*The truth is that you see... erh... Sometimes Support from my friends and some family members keeps me. But mostly sleeping helps me a lot to clear my mind. Anytime I see my friends around me I feel okay and sometimes too if I have no one to talk to I just sleep to forget about the pain”.*

(Patient, Male, 59 years)

From the perspective of Avoidance-Focused Coping strategies, the respondent shared their reactions when encountering unpleasant or uncomfortable situations related to their cancer treatment.

*“There have been times when I’ve felt overwhelmed by the treatment process, and I used to shut myself off. I would sleep more than usual and sometimes even avoid thinking about the diagnosis altogether. But as I progressed, I realized that facing these situations head-on is better. I try not to isolate myself and engage in positive activities but sometimes I wish I could avoid the treatment, but my children won’t permit me”*

(Patient, female, 45 years)

The patients accessed coping strategies from multiple sources, including counselling at the hospital, support from friends and family, guidance from a mental health professional, care from nurses, spiritual counselling from their pastor, and the strength found in prayer and faith. A 58-year-old Patient said: “*Oh sometimes the nurses come in and give us general advice. I’ve learned these strategies from conversations with friends and family and the nurses. My pastor also prays for me”.* Another patient also said:

*“I learned a lot of these strategies through counselling sessions at the hospital. Talking to a mental health professional helped me understand the importance of staying connected and engaging in activities that bring me joy. Some strategies, like seeking solace in my faith during religious services, came from within”* (Patient, Male, 52years).

In our exploration of coping strategies within the context of living with cancer, we uncover a spectrum of effectiveness. Some individuals find their condition remains unchanged despite their efforts, while others discover these strategies enable them to endure the challenges they face. Additionally, a subset reports no significant improvement, highlighting the persistent nature of emotional struggles.

Patients had these to say

*“I’ve noticed that when I actively engage in these coping strategies, my mental state improves. I feel more balanced and able to deal with the challenges. Of course, there are times when things still get tough, but overall, these strategies have helped me maintain a positive outlook”* (Patient, female, 45 years).

*“I’ve learned these strategies from conversations with friends and family and sometimes when the nurses and doctors come to check on us they give us advice. My pastor also motivates me though my condition remains the same”* (Patient, Male, 52 years).

### Mental health services and resources available to patients with cancer

Our exploration reveals diverse patient experiences. Patients’ encounters range from counselling sessions to a complete lack of support, with barriers including issues of availability and stigma. Additionally, the absence of community services tailored to mental health needs underscores the need for enhanced support networks. Our study reveals that specialized mental health support is lacking for many cancer patients, as evidenced by statements from a 59-year-old female patient and a 52-year-old male patient and it continues in most of the in-depth interviews we had with the patients. These patients expressed their unfortunate lack of access to specialized mental health assistance. Nevertheless, they also noted the valuable role played by nurses in providing emotional support and encouragement, highlighting the importance of integrating mental health care into cancer treatment to better address patients’ emotional needs. A 59-year-old female patient said:

*“Unfortunately, I haven’t received specialized mental health support. But sometimes the nurses around speak to us to encourage us that we can make it*”.

Another patient also noted:

*“Oh no…. Unfortunately, I haven’t received specialized mental health support”.* (Patient, Male, 52 years).

We further probe by asking the patients the perspective reason not receiving any mental health support. Our findings shed light on the challenges and perspectives surrounding mental health support among cancer patients. Patients expressed various reasons for not receiving specialized mental health support other patients had these to say:

*“I haven’t sought mental health support mainly due to the lack of available services in my area. It’s not easy to find counselling or support groups close to where I live. There’s also a bit of stigma attached to seeking therapy, which makes it harder to open up about my struggles.*

(Patient, Female, 45 years)

The respondent’s statement regarding the type of community services succinctly conveys the absence of available mental health services within their community, shedding light on a significant gap in accessible resources for addressing emotional well-being in their local area. “*There is no mental health support in the community or services available in the community”* (Patient, Male, 72 years).

**“** *In my community, there are no services available for me. It’s always me and my God”*

(Patient, Male, 45 years)

### Mental health services and resources available for patients with cancer

The mental health services and resources available for patients with cancer from the perspective of health professionals. According to the health workers, there are no specific mental health services available for a patient with cancer but rather they do general counselling at the ward by motivating the patient to cope with their condition and make adherence to their medication. For instance, senior staff nurses noted: From the perspective of the health personnel, The effectiveness of current services appears to contribute to improved patient satisfaction, although they may not be considered unique and are not explicitly linked to survival rates. A 34-year female senior staff nurse health worker noted that:

*“The services are quite effective in terms of improving patient satisfaction, quality of life but not that much as compared to what qualified mental Health officer will do”.*

A 42-year senior staff nurse also said:

*“The services are quite effective in improving patient survival rates. At least some of them can see improvement and cop small with their condition”.*

Health professionals acknowledge the strengths of personalized care and support but emphasize the pressing need for the expansion of mental health services due to low awareness and accessibility issues. A staff burse noted: *“The strengths include personalized care and support. Limitations include the need for wider awareness and accessibility*” Senior Staff Nurse, female 42 years,

*“We don’t have mental health service for those patients in this ward but we the nurses and doctors in charge encourage the patients in the form of general counselling by motivating them”. (*Senior Staff Nurse, female 34 years)

The study further probed for ongoing efforts or plans to enhance or expand mental health services for patients with cancer in the future. A Senior Staff Nurse, female 42 years stated:

*“Erh mm erh……. I don’t think we have ongoing efforts or plans for mental service for cancer patients. We just give them general advice while they are on their bed”.*

In respect to the perspectives of health workers on coping methods used by cancer patients to overcome mental health issues associated with living with cancer showed a spectrum of efficacy. According to the health staff, some patients may believe that their condition stays unchanged despite their proactive efforts, while others believe that these techniques enable them to overcome the problems they face.

A 42-year senior staff nurse said:

*female 42 years) while another health worker also said: Oh. it is like most of them seek support from friends and family*” while A 34-year female senior staff nurse health worker said: *“I think most patients always seek support from friends and family and I think that is the best way they should go”.*

We further ask the health workers how their patient typically reacts when she or he encounters situations related to his or her cancer treatment that he or she finds unpleasant or uncomfortable.

*“Please erh… you see sometimes the patient tends to withdraw especially with the treatment, it is very painful, to be honest.”*

(Senior Staff Nurse, female 34 years).

We also asked questions on the sources of coping strategies adopted by their patient in dealing with their condition. A health worker stated:

*“They learned these coping strategies generally through counselling or the motivation we mostly give in the morning sessions at our facility”.*

(Senior Staff Nurse, female 42 years) while another health worker also said:

*“Through counselling sessions through the nurses around and some of them too their family is doing well to help them”*

*(*Senior Staff Nurse, female 34 years)

## Discussion

This qualitative study explored the lived experiences and coping strategies of cancer patients at Ho Teaching Hospital. Utilizing the Interactive Stress Theory as a conceptual framework, we aimed to understand how a cancer diagnosis and treatment influence psychological well-being and coping mechanisms. In line with the Interactive Stress Theory, our findings highlight the central role of environmental and situational factors in shaping the emotional experiences and coping strategies of cancer patients. For instance, social support, categorized as an environmental factor, emerged as a key source of strength for many patients. This supports the theory’s assertion that a robust support system can buffer against stress and mitigate feelings of isolation. Additionally, spiritual beliefs and practices, identified as critical situational factors, were shown to influence emotional well-being and provide resilience [14]. This further reinforces the theory’s emphasis on the influence of personal and environmental factors on coping strategies. By considering these factors within the Interactive Stress Theory framework, we gain a deeper understanding of how cancer patients navigate their emotional challenges and coping mechanisms.

Our findings reveal that the initial emotional responses to a cancer diagnosis often involve significant distress and devastation. Consistent with existing literature, patients frequently report experiencing shock, fear, anxiety, uncertainty, and hopelessness upon diagnosis [14]. However, the emotional impact is not universally negative; some patients describe positive outcomes, such as increased openness and strengthened relationships following their diagnosis [14]. These varied reactions highlight the complex nature of psychological responses to cancer, influenced by individual factors like resilience [14]. Highly resilient patients may experience levels of anxiety, depression, and happiness similar to those of healthy individuals, demonstrating the variability in emotional responses [15,16]. The study aligns with previous research indicating that cancer survivors often face persistent emotional challenges throughout their cancer journey and beyond. Depression, anxiety, and stress are common among these patients [17,18].

Notably, while many cancer patients experience psychological distress, most do not meet the criteria for specific mental disorders [19]. Despite this, emotional difficulties can significantly affect their quality of life and overall adjustment to the disease. Anxiety tends to increase or worsen at critical stages of the illness, such as diagnosis, treatment initiation and conclusion, recurrence, and terminal stages [19].

Our findings indicate that cancer patients employ a range of coping strategies to manage the challenges associated with their diagnosis and treatment. These strategies include emotional, problem-focused, and avoidance-focused approaches [20]. Previous studies confirm that patients use both adaptive and maladaptive coping mechanisms [20,21]. Frequently observed adaptive strategies include acceptance, seeking emotional and instrumental support, positive reframing, religious coping, and active coping [20,21]. Among these, acceptance emerges as a particularly common strategy [20,21]. Both problem-focused and emotion-focused coping approaches are utilized, with emotion-focused strategies being more prevalent in some cases [22].

Furthermore, strategies such as maintaining a positive outlook, seeking normalcy, relying on family support, and engaging in religious or fatalistic attitudes have also been reported [23]. While our study did not specifically examine the role of healthcare professionals in mitigating suicidal behaviour, it highlighted that cancer patients derive coping strategies from various sources, including healthcare providers, family, friends, and spirituality. In the context of Interactive Stress Theory, these sources can be categorized as environmental factors. A robust social support system can alleviate feelings of loneliness and social isolation, which are central to the theory. The role of spirituality and religious beliefs as sources of strength and resilience among cancer patients aligns with the situational factors outlined in the theory. Spiritual beliefs and practices can influence an individual’s emotional well-being and response to treatment [23]. Our study also underscores the importance of addressing stigma and cultural sensitivity in mental health support. Stigma, a significant situational factor, can discourage individuals from seeking help, reflecting the theory’s emphasis on situational stressors [23].

Additionally, the need for a culturally sensitive approach in mental health care emphasizes the importance of considering cultural norms and beliefs. Integrating mental health support into cancer care is crucial for addressing the emotional needs of patients [23]. Efforts to raise awareness and promote the use of available mental health resources are essential to ensure that individuals facing cancer’s emotional challenges receive the necessary support. Healthcare professionals reported a gap in specialized mental health services for cancer patients, with patients receiving general counselling focused on motivation and coping [24]. This finding reflects the broader issue of limited access to mental health services. Research indicates that cancer survivors often require ongoing care and support [24,25], and preferences for increased access to allied healthcare, including psychology and physiotherapy, are evident [24]. Disparities in access to mental health services, influenced by factors such as race, socioeconomic status, and cancer stage, further complicate the issue [26]. In Ghana, these disparities are compounded by the lack of specialized mental health services within cancer care, making it imperative for the Ghana Health Service and Ministry of Health to prioritize mental health integration within cancer care programs. This includes training healthcare providers to recognize and address emotional distress among cancer patients, creating awareness about mental health resources, and eliminating barriers to care.

In summary, our study provides valuable insights into the emotional experiences and coping strategies of cancer patients. By incorporating the Interactive Stress Theory, we gain a more comprehensive understanding of the psychological responses to cancer and the coping mechanisms employed by patients. Addressing the gaps in mental health services and integrating culturally sensitive approaches can significantly enhance the support provided to cancer patients, ultimately improving their emotional well-being and overall quality of life.

## Conclusion

Cancer patients face profound emotional and psychological challenges, underscoring the urgent need for integrated mental health support in oncology care. This study highlights key areas requiring intervention, including stigma, inadequate mental health services, and limited access to specialized care. To address these gaps, the Ghana Health Service and the Ministry of Health should implement structured mental health screening as part of routine oncology care, ensuring early detection and intervention for psychological distress. Additionally, training healthcare providers in psychosocial support will enhance their ability to identify and manage emotional distress among cancer patients. Establishing dedicated counselling services within oncology units, peer support programs, and telehealth-based mental health services can further improve access to care, particularly in resource-limited settings. Expanding mental health funding and recruiting specialized professionals will also be critical in strengthening support systems for cancer patients. These findings have direct implications for health policy and clinical practice. Integrating mental health into cancer care can improve treatment adherence, enhance quality of life, and reduce long-term psychological burden. Policymakers should leverage these insights to develop targeted interventions, including culturally sensitive mental health programs and nationwide awareness campaigns to reduce stigma. By prioritizing these strategies, Ghana can make significant strides toward achieving the Sustainable Development Goals (SDG 3: Good Health and Well-being and SDG 10: Reduced Inequalities) and ensuring holistic cancer care for all patients.

## Strengths and limitations of the study

The study’s qualitative design provides in-depth insights into the experiences of cancer patients and healthcare professionals. By exploring the emotional and psychological challenges faced by cancer patients, particularly regarding suicidal behaviour and coping strategies, the study offers a nuanced understanding of these issues that are often difficult to capture through quantitative methods. A key strength of this study is the inclusion of multiple perspectives from both cancer patients and healthcare professionals. This dual approach allows for a more comprehensive exploration of the topic, capturing the complexity of the experiences, beliefs, and practices related to mental health and suicidal behaviour in cancer care.

The study was conducted at a single hospital Ho Teaching Hospital limiting its generalizability to other healthcare settings. While the findings offer a detailed understanding of the situation in this specific context, they may not fully reflect the experiences of cancer patients in other regions or healthcare systems, especially in countries with different healthcare infrastructures. The sample size (20 cancer patients and 9 healthcare professionals) was relatively small, which may limit the study’s ability to generalize its findings to larger populations. Though data saturation was achieved, a larger sample size could have provided a broader range of experiences and insights. The study relied on voluntary participation, which may have introduced response bias. Some patients and healthcare professionals declined to participate due to health conditions or time constraints. This may have resulted in a sample that is not fully representative of the broader population, as those with more severe conditions or time constraints may have been underrepresented. Additionally, the study primarily explores the context of Ho Teaching Hospital, which may not fully represent broader healthcare contexts and regional variations in mental health support for cancer patients. The absence of longitudinal data prevents the examination of coping strategy effectiveness over time.

## Supporting information

S1 File This file contains the in-depth interview guides used for data collection in the study, including separate guides for cancer patients and healthcare professionals. The instrument was designed to capture detailed insights into the emotional experiences, coping strategies, and mental health service availability for cancer patients.
(DOCX)
