# Supplementary material for: Lived experiences and coping strategies among cancer patients in the Volta Region of Ghana: A health facility-based qualitative study
Source: PLOS Ment Health. 2025 Apr 30;2(4):e0000310. doi: 10.1371/journal.pmen.0000310 (PMC12798174; doi:10.1371/journal.pmen.0000310)
Supplement: S1 File — This file contains the in-depth interview guides used for data collection in the study, including separate guides for cancer patients and healthcare professionals. The instrument was designed to capture detailed insights into the emotional experiences, coping strategies, and mental health service availability for cancer patients. (DOCX) [file pmen.0000310.s001.docx]

## **RESEARCH INSTRUMENT**

THE UNIVERSITY OF HEALTH AND ALLIED SCIENCES

FRED NEWTON. BINKA SCHOOL OF PUBLIC HEALTH, HOHOE

DEPARTMENT OF POPULATION AND BEHAVIOURIAL SCIENCE

TOPIC**: Lived Experiences and Coping Strategies among Cancer Patients in the Volta Region of Ghana: A Health Facility-Based Qualitative Study**

Dear Participants,

This research is being conducted by a student from the University of Health and Allied Sciences, Fred Newton Binka School of Public Health- Hohoe. The purpose of this study is to explore the Lived Experiences and Coping Strategies among Cancer Patients in the Volta Region of Ghana: A Health Facility-Based Qualitative Study. We, therefore, encourage you to kindly respond to the questions with appropriate and valid answers to ensure accuracy and correct representation. No one will ask you to write your name on any part of the questionnaire. Data analysis will group all participants to protect anonymity, and the findings will be used to explore Lived Experiences and Coping Strategies among Cancer Patients in the Volta Region of Ghana

Your confidentiality and anonymity are assured in this study. Please if you agree to participate, please answer the questions on this survey.

Thank you

## **In-depth interview guide for patients**

**Questions for cancer patients**

Interview number: _____________________________

Date of interview: ______/______/________

Time of interview: Start_________ End ________

Interviewer: ______________________________

**Section A – Demographics characteristics**

**1.** Please what is your age?

2. What is your Sex?

3. Please what is your religion?

4. Please what ethnic group do you belong to?

5. What is your marital status?

6. Please what is your educational level?

7. Please what is your employment status?

8. Please do you have a vailed health insurance?

9. Please where do you reside**?**

### **Section B: LIVED EXPERIENCES OF CANCER PATIENTS**

1. **How did you feel when you first received the cancer diagnosis?**

*(Probe: suicidal ideation, suicidal plan, suicidal attempt)*

1. **What are the most significant emotional challenges you have faced since being diagnosed with cancer?**

**(***Probe: depressed, stress, anxiety,)*

1. **How has your emotional state changed since the diagnosis?**

*(Probe: I felt a mix of shock, fear, and sadness upon hearing the news, feel like a burden to others, feel helpless about my situation, there is no solution to my problem, no one cares about you)*

1. **What emotions do you experience most frequently during your cancer journey?**

**(***Probe: fear, anxiety, hope. Amidst the fear and anxiety, frustration and sadness)*

**SECTION D: COPING STRATEGIES ADOPTED BY CANCER PATIENTS IN DEALING WITH MENTAL HEALTH ISSUES THAT COME WITH LIVING WITH CANCER**

1. **How do you cope with the challenges associated with your condition?**

*(Probe: emotionally focused coping; support from friends, and family, immersing yourself in work to ‘kill time”, self-regulating activities such as reading, listening to music, and watching movies)*

1. **How do you typically react when you encounter situations related to your cancer treatment that you find unpleasant or uncomfortable?**

(*probe: avoidance-focused coping: Denial or avoidance of the diagnosis or treatment, sleeping, using drugs or alcohol, giving up hope for the future, isolation from friends and family)*

1. **What source did you learn these strategies from?** *(Probe; counseling session, religious service, health professionals, etc)*
2. **How successful have these coping strategies been for you?** *(Probe; improvement in condition, or condition remaining same/ retrogressing)*
3. **If one thing could be changed or done to improve the management of your condition, what should that be?**
4. Who do you think should do what you suggested?
5. Is there anything that you feel we should have asked, or that you would like to add?

**SECTION C:** **MENTAL HEALTH SERVICES AND RESOURCES AVAILABLE TO PATIENTS WITH CANCER**

1. **What type of mental health support have you received since you were diagnosed?**

*(Probe; Counseling, Medication, Support groups)*

1. **If you have not received any mental health support, what is the reason for this?**

*(Probe; cost, Lack of available services, stigma)*

1. **In your community what types of resources or services are available for patient living** with cancer?

(Probe; *Counseling, Medication, Support groups)*

**In-depth interview guide for Health worker**

**Questions for Health workers**

Interview number: _____________________________

Date of interview: ______/______/________

Time of interview: Start_________ End ________

Interviewer: ______________________________

**Section A – Demographics characteristics**

**1.** Please what is your age?

2. What is your Sex?

3. Please what is your religion?

4. Please what ethnic group do you belong to?

5. What is your marital status?

6. Please what is your educational level?

7. Please what is your rank?

9. How long have you been working as a nurse?

8. Please how long have you been working as a nurse in this Health facility?

9. Please where do you reside**?**

**Section B: Mental Health Services and Resources Available for Patients with Cancer**

1. What are the main mental health service available to patients with cancers? (Probe: Psychotherapy/Counseling, Support Groups, Integrative Therapies, Psychiatric Medication, Palliative Care)
2. How effective are the services available to patients with cancers (Probe: Improved survival rates, Patient satisfaction, quality of life measure? How do they differ from traditional mental health services, and what makes them effective in addressing the unique mental health needs of patients with cancer)?
3. In your opinion, what are the strengths and limitations of the current mental health services and resources available to patients with cancer?(Probe: identify any specific areas where the services excel or any areas that may require improvement or further attention)?
4. What are the ongoing efforts or plans to enhance or expand mental health services for patients with cancer in the future? (Probe: Upcoming initiatives or strategies in place to further develop or improve mental health support for patients with cancer)?
5. Do you have any recommendations or suggestions for improving?

**Section B: Strategies adopted by cancer patients in dealing with mental health issues that come with living with cancer**

1. **How does your patient cope with the challenges associated with his or her condition?**

*(Probe: emotionally focused coping; support from friends, and family, immersing yourself in work to ‘kill time”, self-regulating activities such as reading, listening to music, and watching movies)*

1. **How did your patient typically react when she or he encounters situations related to his or her cancer treatment that he or she finds unpleasant or uncomfortable?**

(*probe: avoidance-focused coping: Denial or avoidance of the diagnosis or treatment, sleeping, using drugs or alcohol, giving up hope for the future, isolation from friends and family)*

1. **What source did you learn these strategies from?** *(Probe; counseling session, religious, workshop training, school, service, health professionals, etc)*
2. **How successful have these coping strategies been for your patient?** *(Probe; improvement in condition, or condition remaining same/ retrogressing)*
3. **If one thing could be changed or done to improve the management of your condition, what should that be?**
4. Who do you think should do what you suggested?
5. Is there anything that you feel we should have asked, or that you would like to add?
